# Whole brain grey matter synaptic terminal density, age and intellectual functioning in schizophrenia: an in vivo [^11^C]UCB-J positron emission tomography study

**DOI:** 10.1038/s41386-026-02349-7

**Published:** 2026-02-03

**Authors:** Ellis Chika Onwordi, Thomas Whitehurst, Ekaterina Shatalina, Ayla Mansur, Atheeshaan Arumuham, Martin Osugo, Tiago Reis Marques, Sameer Jauhar, Ravi Mehrotra, Maja Ranger, Nikola Rahaman, Steve M. Church, Eugenii A. Rabiner, Roger N. Gunn, Sridhar Natesan, Abraham Reichenberg, Oliver D. Howes

**Affiliations:** 1 Department of Psychosis Studies, Institute of Psychiatry, Psychology & Neuroscience, King’s College London, London, UK; 2 MRC Laboratory of Medical Sciences, Imperial College London, London, UK; 3 South London and Maudsley NHS Foundation Trust, Camberwell, London, UK; 4 East London NHS Foundation Trust, London, UK; 5 IQVIA LTD, London, UK; 6 Division of Psychiatry, Imperial College London, London, UK; 7 Lakeside Unit, West Middlesex University Hospital, West London NHS Trust, London, UK; 8 Westminster Community Rehabilitation Team & Bluebell Lodge, Central and North West London NHS Foundation Trust, London, UK; 9 Kensington Chelsea and Westminster Early Intervention Service, Central and North West London NHS Foundation Trust, London, UK; 10 Invicro, Burlington Danes Building, London, UK; 11 Centre for Neuroimaging Sciences, Institute of Psychiatry, Psychology and Neuroscience, King’s College London, London, UK; 12 Department of Brain Sciences, Imperial College London, The Commonwealth Building, Hammersmith Hospital, London, UK; 13 Department of Psychiatry, Icahn School of Medicine at Mount Sinai, New York, NY USA; 14 Department of Environmental Medicine and Public Health, Icahn School of Medicine at Mount Sinai, New York, NY USA

**Keywords:** Cognitive neuroscience, Schizophrenia

## Abstract

Converging lines of evidence implicate synaptic loss in cognitive impairment associated with schizophrenia. However, it remains unknown whether synaptic terminal density and premorbid intellectual functioning are related in vivo, or whether there are age-related changes in them in schizophrenia. To address this, we investigated whole brain grey matter synaptic vesicle glycoprotein 2A (SV2A) levels and examined their relationship with intellectual functioning and age, in forty-three patients with schizophrenia (SCZ) and 26 healthy volunteers (HV), using [^11^C]UCB-J positron emission tomography (PET). Whole brain grey matter [^11^C]UCB-J distribution volume ratio (DVR) was significantly lower in the SCZ than the HV group (Cohen’s *d* = 0.64, *p* = 0.01), and negatively correlated with age in both groups (Spearman’s rho = –0.46 to –0.55), with no significant group difference in magnitude of DVR-age correlations (*z* = 0.44, *p* = 0.66). Current (Cohen’s *d* = 0.73) and premorbid IQ (Cliff’s delta = 0.37) were significantly lower in the SCZ than the HV group, though DVR was not significantly associated with current or premorbid IQ in either group (including in chronic medicated and early-course unmedicated SCZ subgroups). The group differences in DVR are consistent with a global deficit in synaptic terminal density in schizophrenia, with similar age-related changes in people with schizophrenia and healthy volunteers. The lack of significant relationships between DVR and premorbid or current cognitive measures are not consistent with the hypothesis that lower levels of synaptic terminal density observed in schizophrenia underlie lower levels of intellectual functioning in the disorder.

## Introduction

Schizophrenia is a debilitating illness with a lifetime prevalence of approximately 0.5% [1] and is a leading cause of global disease burden [2]. Its symptoms are often separated into 3 domains: positive (e.g. hallucinations and delusions), negative (e.g. avolition, asociality and anhedonia) and cognitive (e.g. deficits in IQ, executive function, processing speed and working memory) [2, 3]. The modulation of dopamine function is the mainstay of most antipsychotic drugs, which show efficacy in reducing positive symptom severity [4], yet show little benefit in the treatment of cognitive symptoms [5, 6].

Cognitive impairment is present in over 60% of patients at illness onset [7–10], and is linked to worse functional outcomes in schizophrenia [11–13]. In many patients, intellectual functioning is impaired even at the premorbid stage of illness [7, 14–16], and cognitive impairment may progress in some patients through the illness course [17–19], although it is challenging to predict in whom. Together, this highlights the need to advance understanding of the neurobiology of cognitive impairment in schizophrenia.

Several lines of evidence indicate that lower synaptic levels could underlie cognitive impairment associated with schizophrenia [20]. For example, in preclinical models of schizophrenia risk factors using maternal immune activation, offspring of mice exposed to polyriboinosinic-polyribocytidylic acid show lower dendritic spine length and dendritic complexity, coupled with impaired short-term memory in a novel object recognition task, compared to control mice [21]. Furthermore, rodents subjected to another schizophrenia risk factor, chronic stress, show impairments in dendritic arborisation in the medial prefrontal cortex, linked to impaired performance on a perceptual attentional set-shifting task [22]. Moreover, elevated expression of complement component C4, a key regulator of microglia-mediated synaptic elimination [23] that has been strongly associated with schizophrenia risk [24], is linked to lower levels of markers of synaptic density and working memory deficits in mice [25, 26].

There is also growing evidence implicating synaptic dysfunction in schizophrenia pathogenesis [20, 27, 28]. Genetic studies have identified associations between schizophrenia and variants in genes encoding synaptic proteins and protein mediators of synaptic elimination [24, 29, 30]. Post-mortem studies have identified lower levels of synaptic proteins and mRNA [31] and lower dendritic spine density [32, 33] in regions spanning the frontal, temporal and cingulate cortices, and hippocampus in people with schizophrenia relative to healthy volunteers.

Synaptic vesicle glycoprotein 2A (SV2A) is a protein expressed in synaptic terminals, and can be indexed in vivo using positron emission tomography (PET) as a marker of synaptic terminal density [34]. PET studies have identified lower levels of SV2A tracer binding in multiple brain regions than healthy volunteers both in patients with chronic schizophrenia [35, 36] and also in patients early in the course of illness [37–39], although possibly to a lesser extent [40]. Moreover, clinical high-risk subjects have shown lower SV2A binding compared to controls in the anterior cingulate cortex and limbic and associative striatal subdivisions, indicating lower synaptic terminal density in the premorbid phase of illness [39].

Previous work exploring the link between SV2A and cognition has indicated that [^11^C]UCB-J uptake is linked to social cognition and processing speed measures in patients with chronic schizophrenia (*n* = 13 [36]), and with a composite cognitive score in a mixed sample of early-course schizophrenia patients and healthy volunteers (*n* = 18 [37]). Another study of healthy volunteers demonstrated an association between [^11^C]UCB-J DVR and some cognitive functions (e.g. switch cost) but not others (e.g. working memory task performance) [41].

However, no study has tested the association between SV2A and premorbid intellectual functioning or change in intellectual functioning, and only one small study has assessed the relationship with a global measure of current intellectual functioning [37]. Moreover, it remains unknown whether there are age-related changes in SV2A levels in schizophrenia.

Therefore, we conducted an [^11^C]UCB-J PET imaging study to test the relationship between the synaptic terminal density marker SV2A, age and measures of premorbid and current intellectual functioning. We hypothesised that whole brain grey matter SV2A levels would be significantly lower in patients with schizophrenia compared to healthy volunteers, negatively correlated with age, and positively correlated with premorbid and current intellectual functioning in patients with schizophrenia.

## Materials and methods

We obtained approvals for the study protocol from The London-West London & GTAC Research Ethics Committee, United Kingdom (reference: 16/LO/1941), and for the administration of radioactive material from the Administration of Radioactive Substances Advisory Committee, United Kingdom. The study was conducted in accordance with the Declaration of Helsinki (1996). All volunteers provided written informed consent prior to their participation in the study.

We recruited 69 subjects (43 patients with schizophrenia [SCZ] and 26 healthy volunteers [HV]). The whole brain grey matter PET data have not been reported previously for either group. NART and WAIS data have been reported previously for 19  HVs [42]. Volunteers were included in the study if they showed capacity to consent, were 18–65 years of age, and had a normal blood coagulation test result to enable arterial blood sampling.

We recruited patients from first-episode psychosis and general mental health services in London, United Kingdom. Patients with SCZ were included if they met DSM-5 criteria for schizophrenia [43] and had undergone no changes to treatment in the four weeks prior to screening. See Supplementary Materials and Methods for exclusion criteria.

### Clinical assessments

The Structured Clinical Interview for DSM-5 was used to confirm the diagnosis in patients, and to exclude psychiatric illness in healthy volunteers [44]. HVs were also screened for family history of psychosis. In patients, illness duration was calculated as the time from first psychotic symptoms [45].

### Cognitive measures

We used an abbreviated version of the Wechsler Adult Intelligence Scale, shortened version (WAIS-IV SF) to measure current intellectual functioning [46, 47]. The version used in this study incorporated four subtests: digit symbol coding, arithmetic, information, and block design. The National Adult Reading Test (NART) was used to measure premorbid intelligence [48]. These scales were administered and scored in accordance with instructions to generate current (WAIS-IQ) and premorbid IQ (NART-IQ) estimates. See Supplementary Material and Methods for further information.

### Magnetic resonance imaging

To facilitate the delineation of anatomical regions of interest (ROIs), each subject underwent structural magnetic resonance imaging (MRI). See Supplementary Material and Methods for further information.

### PET acquisition

#### [^11^C]UCB-J PET imaging

A low-dose computed tomography scan for attenuation and scatter correction was administered immediately prior to each PET scan. Each subject then received an [^11^C]UCB-J microdose ( ≤ 300 MBq) injected as a smooth bolus via an intravenous cannula (20 mL over 20 s). PET-CT data were acquired for 90 min using a Biograph 6 HiRez PET-CT scanner (Siemens).

### Arterial blood sampling

Arterial blood sampling was performed throughout each PET scan to enable estimation of an arterial input function [49]. See Supplementary Material and Methods for further information.

### Image analysis

We undertook processing and modelling using MIAKAT version 4.3.7 (http://www.miakat.org/MIAKAT2/index.html), implemented in MATLAB (version R2018b; The MathWorks, Inc.) using functions from SPM12 (Wellcome Trust Centre for Neuroimaging, http://www.fil.ion.ucl.ac.uk/spm) and FSL version 5.0.10 (FMRIB). Brain extraction using FSL, and grey matter segmentation and rigid-body coregistration to a standard reference space [50] using SPM12, as implemented via MIAKAT, was applied to each MRI. Next, the template brain image and related Clinical Imaging Centre atlas [51] were warped nonlinearly to the subject’s MRI where the whole brain grey matter was defined as the primary ROI. We used whole brain grey matter as our primary ROI given the evidence from structural and functional neuroimaging studies indicating associations between general intellectual functioning and distributed neural systems rather than single ROIs [52–54], and given evidence for lower [^11^C]UCB-J uptake in multiple brain regions in schizophrenia compared to healthy volunteers [35–37, 40]. We defined frontal and temporal lobe grey matter as additional ROIs for exploratory analyses, given previous findings that the Brief Assessment of Cognition in Schizophrenia (BACS) composite score, a global intellectual functioning measure, is significantly correlated with [^11^C]UCB-J binding in frontal and temporal regions in a combined group of healthy volunteers and patients with schizophrenia [37]. We used the automated anatomical labelling template [55] to generate the centrum semiovale (CS) ROI in line with predefined parameters for its application as a reference region for estimating nondisplaceable [^11^C]UCB-J binding [34].

Individual PET images were motion-corrected using frame-to-frame rigid-body registration, with the 14th frame (acquired 9–11 minutes after injection) used as the reference frame. The summed PET image and MRI were co-registered. Time activity curves were generated for each ROI.

Regional time activity curve and arterial input function data were then analysed together with the 1-tissue compartment model, which has been shown to produce reliable [^11^C]UCB-J volume of distribution (*V*_T_) estimates [49, 56]. Whole brain grey matter *V*_T_ was determined by applying a grey matter mask to the whole brain ROI within MIAKAT. Whole brain grey matter distribution volume ratio (DVR) was obtained by using the CS as a pseudoreference region [34, 49], deriving DVR as a ratio of whole brain grey matter *V*_T_ to CS *V*_T_. We used DVR as our primary [^11^C]UCB-J outcome measure. This approach corrects for non-specific tracer uptake in a reference region, and is thought more closely to reflect the signal specific to SV2A in the ROI than *V*_T_ [57], which indexes both SV2A-specific [^11^C]UCB-J binding, as well as the nondisplaceable uptake. Moreover, the increased variability of [^11^C]UCB-J *V*_T_ compared to DVR [49] means it has lower sensitivity to group differences [58], as found in previous [^11^C]UCB-J analyses [35, 40].

### Sample size and power calculation

We conducted a power calculation using G∗power version 3.1.9.3 (https://www.psychologie.hhu.de/arbeitsgruppen/allgemeine-psychologie-und-arbeitspsychologie/gpower) to establish the minimum sample size required to test our primary hypothesis. This was based on prior evidence for significant correlations between frontal [^11^C]UCB-J binding potential and detection speed (*r* = −0.73, *p* = 0.005) and social emotional cognition domains of the CogState Battery (*r* = 0.64) [36]. We determined that 19 subjects per group would have greater than 80% power to detect a significant relationship at *r* of 0.6 between these variables, at *p* < 0.05 (two-tailed).

### Statistical analysis

Statistical analyses were undertaken with IBM SPSS Statistics, Version 31.0.0.0 (117), and RStudio Version 1.4.1106 (RStudio Team (2021), RStudio, Inc., Boston, MA (http://www.rstudio.com/)).

We used the Kolmogorov-Smirnov test to test data normality (Supplementary Results). We tested relationships between variables using Spearman’s rank correlation coefficient. Group differences in clinical, demographic and imaging variables were assessed using two-tailed independent sample *t* tests for normally distributed data, Mann–Whitney U tests for non-normally distributed data and Chi-squared tests for categorical data. Our primary analysis tested for group differences in whole brain grey matter [^11^C]UCB-J DVR, and for its association with age and WAIS-IQ, in the combined sample, with an uncorrected alpha threshold set at 0.05. We conducted exploratory analyses testing these associations in HV and SCZ groups separately, associations between whole brain grey matter [^11^C]UCB-J DVR and NART-IQ, NART-WAIS IQ difference score (defined as NART-IQ minus WAIS-IQ as a measure of change in intellectual functioning) and WAIS subtests, and for group differences in WAIS-IQ, NART-IQ, and NART-WAIS IQ difference score. We conducted exploratory analyses testing the association between intellectual functioning measures and [^11^C]UCB-J DVR in frontal and temporal regions. Exploratory analyses were conducted using a false discovery rate (FDR) (Q) of 5% to limit false discoveries [59], and FDR-adjusted *p* values are reported.

## Results

Sixty-nine participants (HV *n* = 26 [3 female and 23 male]; SCZ *n* = 43 [7 female and 36 male]) completed the study. In the SCZ group, 20 patients were taking antipsychotic medication (mean [SEM] illness duration = 17.10 [2.63] years) whilst 23 patients were unmedicated (mean [SEM] illness duration = 3.22 [0.65]). The groups were well matched by age and sex, and there were no significant group differences in [^11^C]UCB-J plasma-free fraction (*f*_p_) or CS *V*_T_ (Table 1). [^11^C]UCB-J injected activity was significantly lower in the SCZ group (Table 1).

**Table 1:** Clinico-demographic and imaging characteristics in healthy volunteer (HV) and schizophrenia (SCZ) groups.

	HV (*n* = 26)	SCZ (*n* = 43)	Test-statistic	*p* value
Age (mean [SEM] years)	35.92 (2.45)	33.26 (1.82)	Mann–Whitney U = 483.00	0.35
Female, male (*n*)	3, 23	7, 36	Chi-square = 0.29	0.59
Taking antipsychotic medication (*n*)	–	20	–	–
Duration of illness (mean [SEM] years)	–	9.67 (1.65)	–	–
WAIS-IQ (mean [SEM]) *n*: HV = 20; SCZ = 25	103.43 (3.85)	93.44 (1.97)	T = 2.44	0.02
NART-IQ (mean [SEM]) *n*: HV = 21, SCZ = 33	114.80 (1.55)	107.05 (1.99)	Mann–Whitney U = 217.50	0.02
NART-WAIS IQ difference (NART-IQ minus WAIS-IQ, mean [SEM]) *n*: HV = 19; SCZ = 23	10.34 (3.61)	15.01 (2.51)	T = 1.09	0.28
Injected radioactivity (mean [SEM] MBq)	260.35 (5.35)	228.89 (8.25)	Mann–Whitney U = 366.00	0.02
[^11^C]UCB-J plasma free fraction (mean [SEM])	0.25 (0.005)	0.26 (0.005)	Mann–Whitney U = 658.00	0.22
CS *V*_T_	5.56 (0.11)	6.16 (0.24)	Mann–Whitney U = 675.00	0.15
Whole brain grey matter [^11^C]UCB-J DVR (mean [SEM] ml/cm^3^)	3.43 (0.09)	3.07 (0.09)	T = 2.59	0.01

Sample sizes provided in headings of columns 2 and 3, unless otherwise specified within rows in column 1.

### Current and premorbid intellectual functioning in healthy volunteer and schizophrenia groups

**Fig. 1:**
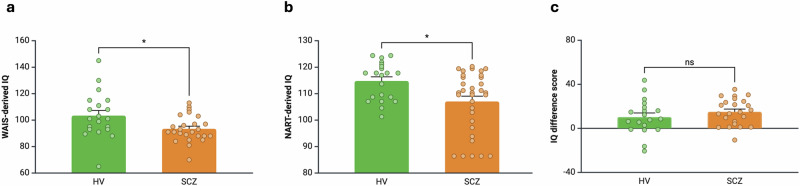
Intellectual functioning measures in healthy volunteer (HV) and schizophrenia (SCZ) groups. **a** WAIS-IQ was significantly lower in the SCZ compared to the HV group (Cohen’s *d* = 0.73). **b** NART-IQ was significantly lower in the SCZ compared to the HV group (Cliff’s delta = 0.37). **c** NART-WAIS IQ difference score was not significantly different between groups. Greater NART-WAIS-IQ difference score reflects greater reduction from premorbid to current IQ. Error bars indicate standard error of the mean.

Mean [SEM] WAIS-IQ was significantly lower in the SCZ (93.33 [2.06]) than the HV group (103.97 [4.02], *t* = 2.36, *p* = 0.02, Cohen’s *d* = 0.73, Table 1, Fig. 1a). Mean [SEM] NART-IQ was significantly lower in SCZ (107.05 [1.99]) than the HV group (114.80 [1.55], Mann–Whitney U = 217.50, *p* = 0.02, Cliff’s delta = 0.37, Table 1, Fig. 1b). There was no significant difference between groups in NART-WAIS IQ difference scores (Table 1, Fig. 1c). Intellectual functioning measures were not significantly associated with chlorpromazine-equivalent antipsychotic dose (Supplementary Results).

### Whole brain grey matter [^11^C]UCB-J DVR in healthy volunteer and schizophrenia groups

**Fig. 2:**
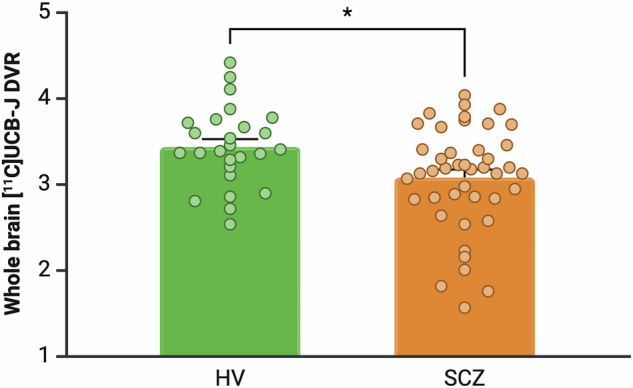
Whole brain grey matter [^11^C]UCB-J DVR levels in healthy volunteer (HV) and schizophrenia (SCZ) groups. [^11^C]UCB-J DVR was significantly lower in the SCZ compared to the HV group (Cohen’s *d* = 0.64). Error bars indicate standard error of the mean.

Mean [SEM] whole brain grey matter [^11^C]UCB-J DVR was significantly lower in SCZ 3.07 [0.09] than the HV group (3.43 [0.09], *t* = 2.59, *p* = 0.01, Cohen’s *d* = 0.64, Fig. 2). There were no significant associations between injected radioactivity and whole brain grey matter [^11^C]UCB-J DVR, and there remained a significant effect of group on whole brain grey matter [^11^C]UCB-J DVR in an ANCOVA controlling for injected radioactivity (*F*_1,66_ = 4.52, *p* = 0.04, η^2^ = 0.06, Supplementary Results). Whole brain grey matter [^11^C]UCB-J DVR was not significantly associated with chlorpromazine-equivalent dose (Supplementary Results). [^11^C]UCB-J DVR remained significantly lower in the SCZ than the HV group in a sensitivity analysis excluding subjects reporting cannabis use in the past month (Supplementary Results).

### Association between whole brain grey matter [^11^C]UCB-J DVR and age

**Fig. 3:**
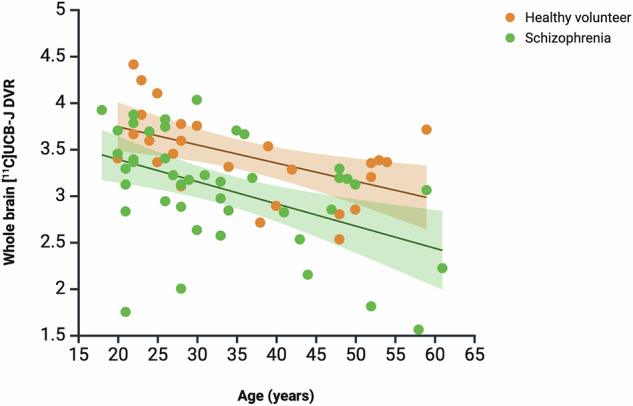
Association between whole brain grey matter [^11^C]UCB-J DVR and age. There was a significant negative relationship between [^11^C]UCB-J DVR and age in the combined sample (rho = -0.45, *p* = 0.0001), HV (rho = –0.55, FDR-corrected *p* = 0.004) and SCZ (rho = -0.45, FDR-corrected *p* = 0.002) groups. Linear regression line shown. Shaded areas indicate 95% confidence interval.

Age was significantly negatively correlated with whole brain grey matter [^11^C]UCB-J DVR in the combined sample (rho = -0.45, *p* = 0.0001, *n* = 69), HV group (rho = -0.55, *p* = 0.004, *n* = 26), and SCZ group (rho = -0.46, *p* = 0.002, *n* = 43, Fig. 3). There was no group difference in the magnitude of the bivariate DVR-age correlations (*z* = 0.44, *p* = 0.66).

### Association between whole brain grey matter [^11^C]UCB-J DVR and general intellectual ability

**Fig. 4:**
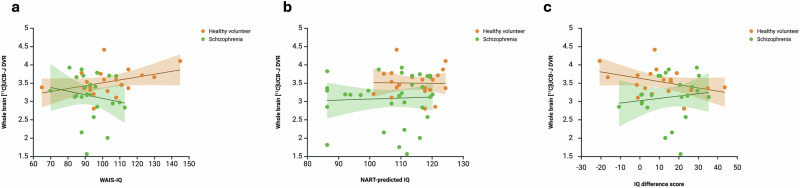
Association between whole brain grey matter [^11^C]UCB-J DVR and intellectual functioning measures. **a** [^11^C]UCB-J DVR was not significantly associated with WAIS-IQ in the combined sample (rho = 0.11, *p* = 0.47), HV (rho = 0.30, FDR-corrected *p* = 0.39) or SCZ (rho = -0.23, FDR-corrected *p* = 0.39) groups. **b** [^11^C]UCB-J DVR was not significantly associated with NART-IQ in the combined sample (rho = 0.15, FDR-corrected *p* = 0.86), HV (rho = 0.04, FDR-corrected *p* = 0.86) or SCZ (rho = 0.06, FDR-corrected *p* = 0.86) groups. **c** [^11^C]UCB-J DVR was not significantly associated with NART-WAIS-IQ difference score in the combined sample (rho = -0.06, FDR-corrected *p* = 0.72), HV (rho = -0.30, FDR-corrected *p* = 0.52) or SCZ (rho = 0.22, FDR-corrected *p* = 0.52) groups. Greater NART-WAIS-IQ difference score reflects greater reduction from premorbid to current IQ. Linear regression lines shown. Shaded areas indicate 95% confidence intervals.

Whole brain grey matter [^11^C]UCB-J DVR was not significantly correlated with WAIS-IQ in the combined sample (rho = 0.11, *p* = 0.47, *n* = 45), or in the HV (rho = 0.30, *p* = 0.39, *n* = 20) or SCZ (rho = -0.23, *p* = 0.39, *n* = 25, Fig. 4a) groups analysed separately. There remained no significant associations between [^11^C]UCB-J DVR and WAIS-IQ when the SCZ group was separated into chronic, medicated patients (rho = -0.07, *p* = 0.86, *n* = 9) and early-course, unmedicated patients (rho = -0.27, *p* = 0.39, *n* = 16).

Whole brain grey matter [^11^C]UCB-J DVR was not significantly correlated with NART-IQ in the combined sample (rho = 0.15, *p* = 0.86, *n* = 54), or in the HV (rho = 0.04, *p* = 0.86, *n* = 21) or SCZ (rho = 0.06, *p* = 0.86, *n* = 33, Fig. 4b) groups. There remained no significant associations between [^11^C]UCB-J DVR and NART-IQ when the SCZ group was separated into chronic, medicated patients (rho = -0.09, *p* = 0.86, *n* = 14) and early-course, unmedicated patients (rho = 0.22, *p* = 0.86, *n* = 19).

Whole brain grey matter [^11^C]UCB-J DVR was not significantly correlated with the NART-WAIS IQ difference score in the combined sample (rho = -0.06, *p* = 0.72, *n* = 42), nor in the HV (rho = -0.30, *p* = 0.52, *n* = 19) or SCZ group (rho = 0.22, *p* = 0.52, *n* = 23, Fig. 4c). There was no significant association between [^11^C]UCB-J DVR and NART-WAIS IQ difference score in the chronic, medicated patients (rho = –0.24, *p* = 0.71, *n* = 8) or early-course, unmedicated patients (rho = 0.52, *p* = 0.23, *n* = 15).

There were no significant associations between whole brain grey matter [^11^C]UCB-J DVR and WAIS subtests (digit symbol coding, arithmetic, block design, or information, Supplementary Table 1).

Further exploratory analyses revealed no significicant associations between intellectual functioning measures and frontal or temporal [^11^C]UCB-J DVR (Supplementary Table 2).

Partial correlations controlling for effects of age and chlorpromazine-equivalent dose revealed no significant associations between [^11^C]UCB-J DVR and intellectual functioning measures (Supplementary Results).

### Association between whole brain grey matter [^11^C]UCB-J DVR and duration of illness

In the SCZ group, duration of illness was not associated with whole brain grey matter [^11^C]UCB-J DVR (rho = –0.21, *p* = 0.72, *n* = 43), WAIS-IQ (rho = –0.02, *p* = 0.94, *n* = 25), NART-IQ (rho = –0.10, *p* = 0.94, *n* = 33), or the NART-WAIS IQ difference score (rho = -0.03, *p* = 0.94, *n* = 23).

Given the correlation between age and SV2A binding in the SCZ group, we conducted partial correlations controlling for the effect of duration of illness in the SCZ group. We found that there remained a significant negative association between age and whole brain grey matter [^11^C]UCB-J DVR in the SCZ group (*r* = -0.35, *p* = 0.02).

## Discussion

Our main findings are that whole brain grey matter [^11^C]UCB-J DVR is significantly negatively associated with age in both schizophrenia and healthy volunteers, but unrelated to measures of current and premorbid intellectual functioning.

Whole brain grey matter [^11^C]UCB-J DVR was lower in schizophrenia compared to controls, as were WAIS-IQ and NART-IQ scores. However, whilst NART-WAIS IQ difference score was numerically greater in the schizophrenia group relative to controls, this was not statistically significant. The DVR finding extends previous work identifying lower SV2A levels in various cortical and subcortical regions in schizophrenia [35–37, 40, 60] by showing for the first time that global grey matter uptake of an SV2A-specific tracer is significantly lower in patients with schizophrenia compared to healthy volunteers. The premorbid and current IQ findings are consistent with meta-analyses finding lower premorbid and current intellectual functioning in schizophrenia compared to controls [14, 15, 17, 61].

In schizophrenia, two previous studies have investigated the associations between [^11^C]UCB-J *BP*_ND_ and specific cognitive functions [36, 37]. The first, analysing 7 a priori ROIs and 21 cognitive measures (147 correlations), reported only two significant positive associations, between frontal *BP*_ND_ and detection speed and social emotional cognition correct responses in CogState Schizophrenia Battery subtests [36]. The second reported significant positive correlations between *BP*_ND_ and BACS composite score in 3 out of 8 regions tested (right rostral middle frontal gyrus, right superior temporal gyrus, and right Heschl’s gyrus) in a combined schizophrenia and healthy control sample [37]. Neither study corrected for multiple comparisons, which increases risk of false positive results [59]. Considering the totality of relationships reported in these studies, the majority (150/155 correlations) were not significant even without multiple comparisons correction. Plausibly, even fewer correlations would have been significant at adjusted probability thresholds if FDR correction had been applied. Our findings add to these studies in a sample two- to threefold larger, providing further evidence that there is not a strong relationship between SV2A markers and generalised cognitive function in schizophrenia or healthy young to middle-aged adults, although weaker associations or associations with specific cognitive functions remain possible. Exploratory analyses did not find significant relationships between DVR and cognitive measures in chronic or first-episode subgroups. However, the sample sizes were small, particularly for the chronic subgroup, and these analyses are underpowered so require further testing in future studies.

We found negative correlations between whole brain grey matter [^11^C]UCB-J DVR and age, in the combined sample, and in healthy control and schizophrenia groups (Spearman’s rho ranging from –0.55 to –0.45). In the schizophrenia group, this relationship was unaffected by duration of illness, indicating it primarily reflects normal developmental rather than disorder-related changes. These findings are consistent with previous work finding negative associations between cortical and subcortical (particularly caudate) SV2A levels as indexed by *V*_T_ and age [49, 62, 63], and smaller effects in the medial thalamus, transverse temporal gyrus, lateral posterior insula and caudate nucleus with standardised uptake value ratio as an alternative SV2A outcome measure [64]. Our findings extend these by identifying brain-wide age-related effects on SV2A levels. Our findings contrast with smaller studies exploring SV2A-age relationships in controls and people with psychosis [35, 36, 65]. Differences between findings may be partially explained by the greater power that our study has to detect SV2A-age relationships.

### Strengths and limitations

To date, this is the largest sample of schizophrenia patients investigated with [^11^C]UCB-J PET imaging. 20 patients were taking antipsychotic medication, which may have influenced the relationship between SV2A binding and intellectual functioning. Antipsychotic drugs may reduce cognitive impairment in schizophrenia [66, 67], although they show heterogeneous effects potentially relating to receptor-binding profiles [68]. Antipsychotic drugs ameliorating cognitive impairment in schizophrenia could weaken the association between synaptic terminal density and cognitive function. However, chlorpromazine-equivalent dose was not significantly correlated with intellectual functioning measures or DVR. Moreover, the absence of significant relationships between DVR and intellectual functioning measures remained consistent when accounting for treatment status, either through analyses of antipsychotic-treated and unmedicated subgroups, or partial correlations controlling for antipsychotic dose. Taken together with prior evidence that SV2A binding in patients is not significantly related to antipsychotic exposure [35–37, 39], and that SV2A levels in the rat brain are unaffected by clinically relevant antipsychotic drug exposure [35], it is unlikely that treatment status accounted for our findings. Nonetheless, further studies including antipsychotic-naive patients would be useful to test if antipsychotic exposure could be confounding our findings.

A small proportion of volunteers reported cannabis use within the last month (healthy volunteer *n* = 1, schizophrenia *n* = 4). This is a potential limitation, but DVR results remained consistent in a sensitivity analysis excluding these volunteers. Thus, recent cannabis use is unlikely to influence our findings. Future studies examining volunteers free from cannabis use would be valuable.

We calculated DVR using the CS as a reference region. A potential limitation is that the CS shows low levels of [^11^C]UCB-J displacement by levetiracetam, an SV2A-specific drug [34]. Thus, group differences in CS *V*_T_ could bias findings. However, we did not find significant differences between schizophrenia and healthy volunteer groups in CS *V*_T_, consistent with prior work [35, 36, 38].

Our study was powered to detect DVR-age and DVR-WAIS-IQ correlations with *r* = 0.6. It was underpowered to detect weaker relationships, or associations in smaller subgroups, so type II error is possible.

We did not set out principally to explore the relationship between SV2A binding and decline in intellectual functioning in schizophrenia, or to include patients showing pronounced decline. Although NART-WAIS IQ difference score was greater in schizophrenia compared with controls suggesting increasing cognitive impairment, this was not statistically significant. This analysis may have been underpowered due to sample size, or more sensitive to measurement error through use of cross-sectional measures to estimate longitudinal change in IQ. Recent cross-sectional and longitudinal MRI studies stratifying patients according to IQ trajectories indicate lower brain volume and greater brain volume loss respectively in subgroups with more negative trajectories versus those with relatively stable IQ [69, 70]. Whether schizophrenia subtypes showing more pronounced decline in intellectual functioning would also show altered SV2A binding relative to stabler subtypes remains unclear. Moreover, a recent meta-analysis of longitudinal studies indicates that, despite broad stability in cognitive functioning following the first psychotic episode, verbal and visual learning and memory domains may decline [17]. Future studies focusing on specific cognitive domains, and in patient groups showing greater cognitive decline, are warranted.

[^11^C]UCB-J DVR, which indexes SV2A binding, a marker of synaptic terminal density [34], was not significantly associated with intellectual functioning measures. Whilst this indicates that alterations in SV2A are unlikely to underlie cognitive impairment in schizophrenia, this does not preclude alterations in other aspects of synaptic function underlying cognitive impairment. Indeed, genetic and postmortem evidence implicates disruption in synaptic parameters additional to terminal density in schizophrenia, including cellular machinery relevant to synaptic plasticity such as *N*-methyl-D-aspartate receptor and activity regulated cytoskeleton-associated protein complexes, dendritic spine density and PSD-95 protein levels [29, 30, 71, 72]. Thus, disorder-related changes in these other synaptic components may underlie cognitive impairment in schizophrenia [73]. The development of in vivo molecular imaging probes to synaptic elements beyond SV2A would be valuable in further testing the link between synaptic dysfunction and cognitive impairment in schizophrenia.

Our previous studies [35, 40] focused on a limited number of a priori ROIs (frontal cortex, anterior cingulate cortex, and hippocampus) selected given meta-analytic findings for lower synaptic protein levels in the postmortem brain in schizophrenia [31], and exploratory ROIs (temporal, parietal, and occipital lobes, dorsolateral prefrontal cortex, thalamus and amygdala) selected given meta-analytic findings for structural and/or functional brain alterations in schizophrenia [74, 75]. Those analyses did not investigate other regions such as the insula, cerebellum, basal ganglia, and brainstem, which may nonetheless have important contributions to differences in brain synaptic terminal density in schizophrenia. Moreover, it has previously been unknown whether schizophrenia-related differences in synaptic terminal density are detectable at the whole-brain level, which has important implications with respect to both the magnitude and distribution of effects, and measurement sensitivity. Structural and functional alterations in regions beyond those considered in our previous analyses have been implicated in impaired cognitive functioning in schizophrenia [76–81]. Given this, and the evidence that general intellectual functioning arises from distributed neural systems rather than discrete regions [52–54], it is important to consider whether synaptic alterations throughout the brain are linked to intellectual functioning in schizophrenia. Nonetheless, there could be differences in SV2A binding in schizophrenia in specific neural circuits spanning multiple regions relevant to intellectual functioning that this approach is unable to detect. However, exploratory analyses did not identify significant associations between intellectual functioning measures and frontal or temporal [^11^C]UCB-J DVR either.

### Implications for understanding the pathophysiology of schizophrenia

Our finding of lower global [^11^C]UCB-J DVR in schizophrenia is consistent with our and other previous findings of lower SV2A-specific radioligand binding in cortical brain regions in schizophrenia [35–37, 40, 60] and extends them to indicate globally lower grey matter levels. One potential explanation for lower SV2A levels in schizophrenia is that these are due to a neurodevelopmental mechanism predating the first psychotic episode, reflected in lower premorbid IQ [82]. Our finding that NART-IQ, whilst lower in schizophrenia, was not significantly associated with DVR, is not consistent with this explanation. Similarly, our finding that WAIS-IQ is not associated with DVR is not consistent with the hypothesis that SV2A levels underlie current cognitive function. However, given the prior findings of regional and test specific relationships, and the fact that we cannot exclude weak relationships, or relationships in patient subgroups, further, particularly longitudinal studies, are required to test these hypotheses further.

Our finding that there is no significant group difference in the magnitude of negative [^11^C]UCB-J DVR-age correlations suggests that age-related decline in presynaptic terminal markers in schizophrenia is similar to that seen in healthy controls. In contrast, previous cross-sectional work has identified accelerated age-related grey matter volume loss in schizophrenia compared to controls until middle age, stabilising thereafter [83], with meta-analyses of longitudinal MRI studies similarly identifiying greater grey matter volume loss over time in patients [84]. Notably, grey matter volume changes are unlikely wholly attributable to synaptic terminal loss, and likely partially attributable to changes in its other consitutents, including vasculature, astrocytes, neuronal somata and dendrites [85, 86]. Longitudinal PET studies exploring synaptic terminal density in larger numbers of younger subjects and clinical high-risk subjects would be useful to address this.

In conclusion, whole brain grey matter [^11^C]UCB-J DVR, a marker of global synaptic terminal density, is lower in schizophrenia, and negatively associated with age, but unrelated to measures of premorbid or current intelligence in healthy volunteers or in patients with schizophrenia. Our findings suggest that synaptic terminal density declines with age at a similar rate in patients and controls, and that factors underlying lower levels of global intellectual functioning in schizophrenia are distinct from those underlying lower synaptic vesicle glycoprotein 2A levels observed in schizophrenia.

## Supplementary information

Whole brain grey matter synaptic terminal density, age and intellectual functioning in schizophrenia: an in vivo [<sup>11</sup>C]UCB-J positron emission tomography study. Supplemental Material

## Data Availability

Imaging and related clinical data will be made available upon reasonable request.

## References

[1] Saha S, Chant D, Welham J, McGrath J. A systematic review of the prevalence of schizophrenia. PLoS Med. 2005;2:e141. 15916472 10.1371/journal.pmed.0020141 PMC1140952

[2] McCutcheon RA, Reis Marques T, Howes OD. Schizophrenia-An Overview. JAMA Psychiatry. 2020;77:201–210. 10.1001/jamapsychiatry.2019.3360 31664453

[3] Jauhar S, Johnstone M, McKenna PJ. Schizophrenia. Lancet. 2022;399:473–86. 35093231 10.1016/S0140-6736(21)01730-X

[4] Kaar SJ, Natesan S, McCutcheon R, Howes OD. Antipsychotics: Mechanisms underlying clinical response and side-effects and novel treatment approaches based on pathophysiology. Neuropharmacology. 2020;172:107704. 10.1016/j.neuropharm.2019.107704 31299229

[5] McCutcheon RA, Keefe RSE, McGuire PK. Cognitive impairment in schizophrenia: aetiology, pathophysiology, and treatment. Mol Psychiatry. 2023;28:1902–18. 36690793 10.1038/s41380-023-01949-9 PMC10575791

[6] Veerman SRT, Schulte PFJ, de Haan L. Treatment for negative symptoms in schizophrenia: a comprehensive review. Drugs. 2017;77:1423–59. 28776162 10.1007/s40265-017-0789-y

[7] Stainton A, Chisholm K, Griffiths SL, Kambeitz-Ilankovic L, Wenzel J, Bonivento C, et al. Prevalence of cognitive impairments and strengths in the early course of psychosis and depression. Psychol Med. 2023;53:5945–57. 37409883 10.1017/S0033291723001770 PMC10520593

[8] Mwesiga EK, Robbins R, Akena D, Koen N, Nakku J, Nakasujja N, et al. Prevalence, profile and associations of cognitive impairment in Ugandan first-episode psychosis patients. Schizophr Res Cogn. 2022;28:100234. 35024348 10.1016/j.scog.2021.100234 PMC8728100

[9] Ugwuonye OK, Onu JU, Iyidobi TO, Ohaeri JU. Prevalence of neurocognitive deficits in patients with first-episode schizophrenia in an African sample and its relationship with dimensions of psychopathology and psychosocial outcome. BMC Psychiatry. 2024;24:866. 39617903 10.1186/s12888-024-06315-9 PMC11610062

[10] Peng XJ, Hei GR, Yang Y, Liu CC, Xiao JM, Long YJ, et al. The association between cognitive deficits and clinical characteristic in first-episode drug naive patients with schizophrenia. Front Psychiatry. 2021;12:638773. 33716832 10.3389/fpsyt.2021.638773 PMC7950319

[11] Fett AK, Viechtbauer W, Dominguez MD, Penn DL, van Os J, Krabbendam L. The relationship between neurocognition and social cognition with functional outcomes in schizophrenia: a meta-analysis. Neurosci Biobehav Rev. 2011;35:573–88. 20620163 10.1016/j.neubiorev.2010.07.001

[12] Halverson TF, Orleans-Pobee M, Merritt C, Sheeran P, Fett AK, Penn DL. Pathways to functional outcomes in schizophrenia spectrum disorders: Meta-analysis of social cognitive and neurocognitive predictors. Neurosci Biobehav Rev. 2019;105:212–9. 31415864 10.1016/j.neubiorev.2019.07.020

[13] Kharawala S, Hastedt C, Podhorna J, Shukla H, Kappelhoff B, Harvey PD. The relationship between cognition and functioning in schizophrenia: a semi-systematic review. Schizophr Res Cogn. 2022;27:100217. 34631435 10.1016/j.scog.2021.100217 PMC8488595

[14] Woodberry KA, Giuliano AJ, Seidman LJ. Premorbid IQ in schizophrenia: a meta-analytic review. Am J Psychiatry. 2008;165:579–87. 18413704 10.1176/appi.ajp.2008.07081242

[15] Khandaker GM, Barnett JH, White IR, Jones PB. A quantitative meta-analysis of population-based studies of premorbid intelligence and schizophrenia. Schizophr Res. 2011;132:220–7. 21764562 10.1016/j.schres.2011.06.017 PMC3485562

[16] Mohn-Haugen CR, Mohn C, Laroi F, Teigset CM, Oie MG, Rund BR. A systematic review of premorbid cognitive functioning and its timing of onset in schizophrenia spectrum disorders. Schizophr Res Cogn. 2022;28:100246. 35251943 10.1016/j.scog.2022.100246 PMC8892142

[17] Catalan A, McCutcheon RA, Aymerich C, Pedruzo B, Radua J, Rodriguez V, et al. The magnitude and variability of neurocognitive performance in first-episode psychosis: a systematic review and meta-analysis of longitudinal studies. Transl Psychiatry. 2024;14:15. 38191534 10.1038/s41398-023-02718-6 PMC10774360

[18] Chan SKW, Liao Y, Hui CLM, Wong TY, Suen Y, Chang WC, et al. Longitudinal changes of cognitive function and its relationship with subdomains of negative symptoms in patients with adult-onset first-episode schizophrenia: a 4-year follow up study. Schizophr Res. 2023;252:181–8. 36657362 10.1016/j.schres.2023.01.004

[19] Jonas K, Lian W, Callahan J, Ruggero CJ, Clouston S, Reichenberg A, et al. The course of general cognitive ability in individuals with psychotic disorders. JAMA Psychiatry. 2022;79:659–66. 35583896 10.1001/jamapsychiatry.2022.1142 PMC9118026

[20] Howes OD, Onwordi EC. The synaptic hypothesis of schizophrenia version III: a master mechanism. Mol Psychiatry. 2023;28:1843–56. 37041418 10.1038/s41380-023-02043-w PMC10575788

[21] Li WY, Chang YC, Lee LJ, Lee LJ. Prenatal infection affects the neuronal architecture and cognitive function in adult mice. Dev Neurosci. 2014;36:359–70. 24942872 10.1159/000362383

[22] Liston C, Miller MM, Goldwater DS, Radley JJ, Rocher AB, Hof PR, et al. Stress-induced alterations in prefrontal cortical dendritic morphology predict selective impairments in perceptual attentional set-shifting. J Neurosci. 2006;26:7870–4. 16870732 10.1523/JNEUROSCI.1184-06.2006 PMC6674229

[23] Stevens B, Allen NJ, Vazquez LE, Howell GR, Christopherson KS, Nouri N, et al. The classical complement cascade mediates CNS synapse elimination. Cell. 2007;131:1164–78. 18083105 10.1016/j.cell.2007.10.036

[24] Sekar A, Bialas AR, de Rivera H, Davis A, Hammond TR, Kamitaki N, et al. Schizophrenia risk from complex variation of complement component 4. Nature. 2016;530:177–83. 26814963 10.1038/nature16549 PMC4752392

[25] Druart M, Nosten-Bertrand M, Poll S, Crux S, Nebeling F, Delhaye C, et al. Elevated expression of complement C4 in the mouse prefrontal cortex causes schizophrenia-associated phenotypes. Mol Psychiatry. 2021;26:3489–501. 33837272 10.1038/s41380-021-01081-6

[26] Yilmaz M, Yalcin E, Presumey J, Aw E, Ma M, Whelan CW, et al. Overexpression of schizophrenia susceptibility factor human complement C4A promotes excessive synaptic loss and behavioral changes in mice. Nat Neurosci. 2021;24:214–24. 33353966 10.1038/s41593-020-00763-8 PMC8086435

[27] Feinberg I. Schizophrenia: caused by a fault in programmed synaptic elimination during adolescence?. J Psychiatr Res. 1982;17:319–34. 7187776 10.1016/0022-3956(82)90038-3

[28] Keshavan MS, Anderson S, Pettergrew JW. Is Schizophrenia due to excessive synaptic pruning in the prefrontal cortex? The Feinberg hypothesis revisited. J Psychiatr Res. 1994;28:239–65. 7932285 10.1016/0022-3956(94)90009-4

[29] Purcell SM, Moran JL, Fromer M, Ruderfer D, Solovieff N, Roussos P, et al. A polygenic burden of rare disruptive mutations in schizophrenia. Nature. 2014;506:185–90. 24463508 10.1038/nature12975 PMC4136494

[30] Fromer M, Pocklington AJ, Kavanagh DH, Williams HJ, Dwyer S, Gormley P, et al. De novo mutations in schizophrenia implicate synaptic networks. Nature. 2014;506:179–84. 24463507 10.1038/nature12929 PMC4237002

[31] Osimo EF, Beck K, Reis Marques T, Howes OD. Synaptic loss in schizophrenia: a meta-analysis and systematic review of synaptic protein and mRNA measures. Mol Psychiatry. 2019;24:549–61. 29511299 10.1038/s41380-018-0041-5 PMC6004314

[32] Garey LJ, Ong WY, Patel TS, Kanani M, Davis A, Mortimer AM, et al. Reduced dendritic spine density on cerebral cortical pyramidal neurons in schizophrenia. J Neurol Neurosurg Psychiatry. 1998;65:446–53. 9771764 10.1136/jnnp.65.4.446 PMC2170311

[33] Glantz LA, Lewis DA. Decreased dendritic spine density on prefrontal cortical pyramidal neurons in schizophrenia. Arch Gen Psychiatry. 2000;57:65–73. 10632234 10.1001/archpsyc.57.1.65

[34] Finnema SJ, Nabulsi NB, Eid T, Detyniecki K, Lin SF, Chen MK, et al. Imaging synaptic density in the living human brain. Sci Transl Med. 2016;8:348ra96. 27440727 10.1126/scitranslmed.aaf6667

[35] Onwordi EC, Halff EF, Whitehurst T, Mansur A, Cotel M-C, Wells L, et al. Synaptic density marker SV2A is reduced in schizophrenia patients and unaffected by antipsychotics in rats. Nat Commun. 2020;11:246. 31937764 10.1038/s41467-019-14122-0 PMC6959348

[36] Radhakrishnan R, Skosnik PD, Ranganathan M, Naganawa M, Toyonaga T, Finnema S, et al. In vivo evidence of lower synaptic vesicle density in schizophrenia. Mol Psychiatry. 2021;26:7690–8. 34135473 10.1038/s41380-021-01184-0

[37] Yoon JH, Zhang Z, Mormino E, Davidzon G, Minzenberg MJ, Ballon J, et al. Reductions in synaptic marker SV2A in early-course Schizophrenia. J Psychiatr Res. 2023;161:213–7. 36934603 10.1016/j.jpsychires.2023.02.026

[38] Onwordi EC, Whitehurst T, Shatalina E, Carr R, Mansur A, Arumuham A, et al. The relationship between cortical synaptic terminal density marker SV2A and glutamate early in the course of schizophrenia: a multimodal PET and MRS imaging study. Transl Psychiatry. 2025;15:70. 40025026 10.1038/s41398-025-03269-8 PMC11873237

[39] Blasco MB, Nisha Aji K, Ramos-Jimenez C, Leppert IR, Tardif CL, Cohen J, et al. Synaptic Density in Early Stages of Psychosis and Clinical High Risk. JAMA Psychiatry. 2025;82:171–80. 39535765 10.1001/jamapsychiatry.2024.3608 PMC11561726

[40] Onwordi EC, Whitehurst T, Shatalina E, Mansur A, Arumuham A, Osugo M, et al. Synaptic Terminal Density Early in the Course of Schizophrenia: an in vivo UCB-J Positron Emission Tomographic Imaging Study of Synaptic Vesicle Glycoprotein 2A (SV2A). LID - S0006-3223(23)01353-7 [pii] LID 10.1016/j.biopsych.2023.05.022 2023(1873-2402 (Electronic)).

[41] Shatalina E, Onwordi EC, Whitehurst T, Whittington A, Mansur A, Arumuham A, et al. The relationship between SV2A levels, neural activity, and cognitive function in healthy humans: a [11C]UCB-J PET and fMRI study. Imaging Neurosci (Camb). 2024;2:1–16. 39989611 10.1162/imag_a_00190 PMC11840333

[42] Shatalina E, Whitehurst TS, Onwordi EC, Gilbert BJ, Rizzo G, Whittington A, et al. Mitochondrial complex I density is associated with IQ and cognition in cognitively healthy adults: an in vivo [(18)F]BCPP-EF PET study. EJNMMI Res. 2024;14:41. 38632153 10.1186/s13550-024-01099-1 PMC11024075

[43] American Psychiatric Association. Schizophrenia Spectrum and Other Psychotic Disorders. Diagnostic and Statistical Manual of Mental Disorders. 5th ed. Washington, DC (2013).

[44] First MB, Williams JBW, Karg RS, Spitzer RL, Structured Clinical Interview for DSM-5—Research Version (SCID-5 for DSM-5, Research Version; SCID-5-RV). (2015).

[45] Beiser M, Erickson D, Fleming JA, Iacono WG. Establishing the onset of psychotic illness. Am J Psychiatry. 1993;150:1349–54. 8352345 10.1176/ajp.150.9.1349

[46] Bulzacka E, Meyers JE, Boyer L, Le Gloahec T, Fond G, Szoke A, et al. WAIS-IV seven-subtest short form: validity and clinical use in schizophrenia. Arch Clin Neuropsychol. 2016;31:915–25. 27590304 10.1093/arclin/acw063

[47] Wechsler D. WAIS-III: Administration and Scoring Manual: Wechsler Adult Intelligence Scale, Psychological Corporation, San Antonio, TX (1997).

[48] Bright P, Jaldow E, Kopelman MD. The National Adult Reading Test as a measure of premorbid intelligence: a comparison with estimates derived from demographic variables. J Int Neuropsychol Soc. 2002;8:847–54. 12240749 10.1017/s1355617702860131

[49] Mansur A, Rabiner EA, Comley RA, et al. Characterization of 3 PET Tracers for Quantification of Mitochondrial and Synaptic Function in Healthy Human Brain: 18F-BCPP-EF, 11C-SA-4503, and 11C-UCB-J. J Nucl Med. 2020;61:96–103. 10.2967/jnumed.119.228080 31324712

[50] Grabner G, Janke AL, Budge MM, Smith D, Pruessner J, Collins DL, editors. Symmetric Atlasing and Model Based Segmentation: An Application to the Hippocampus in Older Adults. Medical Image Computing and Computer-Assisted Intervention – MICCAI 2006; Berlin, Heidelberg: Springer Berlin Heidelberg. 10.1007/11866763_8 17354756

[51] Tziortzi AC, Searle GE, Tzimopoulou S, Salinas C, Beaver JD, Jenkinson M, et al. Imaging dopamine receptors in humans with [11C]-(+)-PHNO: dissection of D3 signal and anatomy. Neuroimage. 2011;54:264–77. 20600980 10.1016/j.neuroimage.2010.06.044

[52] Dubois J, Galdi P, Paul LK, Adolphs R. A distributed brain network predicts general intelligence from resting-state human neuroimaging data. Philos Trans R Soc Lond B Biol Sci. 2018;373:20170284. 10.1098/rstb.2017.0284 PMC6107566 30104429

[53] Anderson ED, Barbey AK. Investigating cognitive neuroscience theories of human intelligence: a connectome-based predictive modeling approach. Hum Brain Mapp. 2023;44:1647–65. 36537816 10.1002/hbm.26164 PMC9921238

[54] Hussain MA, LaMay D, Grant E, Ou Y. Deep learning of structural MRI predicts fluid, crystallized, and general intelligence. Sci Rep. 2024;14:27935. 39537706 10.1038/s41598-024-78157-0 PMC11561325

[55] Tzourio-Mazoyer N, Landeau B, Papathanassiou D, Crivello F, Etard O, Delcroix N, et al. Automated anatomical labeling of activations in SPM using a macroscopic anatomical parcellation of the MNI MRI single-subject brain. NeuroImage. 2002;15:273–89. 11771995 10.1006/nimg.2001.0978

[56] Finnema SJ, Nabulsi NB, Mercier J, Lin SF, Chen MK, Matuskey D, et al. Kinetic evaluation and test-retest reproducibility of [(11)C]UCB-J, a novel radioligand for positron emission tomography imaging of synaptic vesicle glycoprotein 2A in humans. J Cereb Blood Flow Metab. 2018;38:2041–52. 28792356 10.1177/0271678X17724947 PMC6259313

[57] Rossano S, Toyonaga T, Finnema SJ, Naganawa M, Lu Y, Nabulsi N, et al. Assessment of a white matter reference region for (11)C-UCB-J PET quantification. J Cereb Blood Flow Metab. 2019:271678X19879230. 10.1177/0271678X19879230 PMC7446568 31570041

[58] Bloomfield PS, Selvaraj S, Veronese M, Rizzo G, Bertoldo A, Owen DR, et al. Microglial activity in people at ultra high risk of psychosis and in schizophrenia: an [(11)C]PBR28 PET brain imaging study. Am J Psychiatry. 2016;173:44–52. 26472628 10.1176/appi.ajp.2015.14101358 PMC4821370

[59] Benjamini Y, Hochberg Y. Controlling the false discovery rate: a practical and powerful approach to multiple testing. J R Stat Soc Ser B (Methodol). 1995;57:289–300.

[60] Onwordi EC, Whitehurst T, Mansur A, Statton B, Berry A, Quinlan M, et al. The relationship between synaptic density marker SV2A, glutamate and N-acetyl aspartate levels in healthy volunteers and schizophrenia: a multimodal PET and magnetic resonance spectroscopy brain imaging study. Transl Psychiatry. 2021;11:393. 34282130 10.1038/s41398-021-01515-3 PMC8290006

[61] Mesholam-Gately RI, Giuliano AJ, Goff KP, Faraone SV, Seidman LJ. Neurocognition in first-episode schizophrenia: a meta-analytic review. Neuropsychology. 2009;23:315–36. 19413446 10.1037/a0014708

[62] Toyonaga T, Khattar N, Wu Y, Lu Y, Naganawa M, Gallezot JD, et al. The regional pattern of age-related synaptic loss in the human brain differs from gray matter volume loss: in vivo PET measurement with [(11)C]UCB-J. Eur J Nucl Med Mol Imaging. 2024;51:1012–22. 37955791 10.1007/s00259-023-06487-8 PMC12701775

[63] Fang XT, Volpi T, Holmes SE, Esterlis I, Carson RE, Worhunsky PD. Linking resting-state network fluctuations with systems of coherent synaptic density: A multimodal fMRI and (11)C-UCB-J PET study. Front Hum Neurosci. 2023;17:1124254. 36908710 10.3389/fnhum.2023.1124254 PMC9995441

[64] Michiels L, Delva A, van Aalst J, Ceccarini J, Vandenberghe W, Vandenbulcke M, et al. Synaptic density in healthy human aging is not influenced by age or sex: a (11)C-UCB-J PET study. Neuroimage. 2021;232:117877. 33639258 10.1016/j.neuroimage.2021.117877

[65] Andersen KB, Hansen AK, Knudsen K, Schacht AC, Damholdt MF, Brooks DJ, et al. Healthy brain aging assessed with [(18)F]FDG and [(11)C]UCB-J PET. Nucl Med Biol. 2022;112-113:52–8. 35820300 10.1016/j.nucmedbio.2022.06.007

[66] Baldez DP, Biazus TB, Rabelo-da-Ponte FD, Nogaro GP, Martins DS, Kunz M, et al. The effect of antipsychotics on the cognitive performance of individuals with psychotic disorders: Network meta-analyses of randomized controlled trials. Neurosci Biobehav Rev. 2021;126:265–75. 33812977 10.1016/j.neubiorev.2021.03.028

[67] Feber L, Peter NL, Chiocchia V, Schneider-Thoma J, Siafis S, Bighelli I, et al. Antipsychotic Drugs and Cognitive Function: A Systematic Review and Network Meta-Analysis. JAMA Psychiatry. 2025;82:47–56. 39412783 10.1001/jamapsychiatry.2024.2890 PMC11581732

[68] McCutcheon RA, Harrison PJ, Howes OD, McGuire PK, Taylor DM, Pillinger T. Data-Driven Taxonomy for Antipsychotic Medication: A New Classification System. Biol Psychiatry. 2023;94:561–8. 37061079 10.1016/j.biopsych.2023.04.004 PMC10914668

[69] Yamazaki R, Matsumoto J, Ito S, Nemoto K, Fukunaga M, Hashimoto N, et al. Longitudinal reduction in brain volume in patients with schizophrenia and its association with cognitive function. Neuropsychopharmacol Rep. 2024;44:206–15. 38348613 10.1002/npr2.12423 PMC10932790

[70] Watson AJ, Giordano A, Suckling J, Barnes TRE, Husain N, Jones PB, et al. Cognitive function in early-phase schizophrenia-spectrum disorder: IQ subtypes, brain volume and immune markers. Psychol Med. 2023;53:2842–51. 35177144 10.1017/S0033291721004815 PMC10244009

[71] Marshall CR, Howrigan DP, Merico D, Thiruvahindrapuram B, Wu W, Greer DS, et al. Contribution of copy number variants to schizophrenia from a genome-wide study of 41,321 subjects. Nat Genet. 2017;49:27–35. 27869829 10.1038/ng.3725 PMC5737772

[72] Berdenis van Berlekom A, Muflihah CH, Snijders GJLJ, et al. Synapse Pathology in Schizophrenia: A Meta-analysis of Postsynaptic Elements in Postmortem Brain Studies. Schizophr Bull. 2020;46:374–86. 10.1093/schbul/sbz060 PMC7442385 31192350

[73] Forsyth JK, Lewis DA. Mapping the consequences of impaired synaptic plasticity in schizophrenia through development: an integrative model for diverse clinical features. Trends Cogn Sci. 2017;21:760–78. 28754595 10.1016/j.tics.2017.06.006 PMC5610626

[74] Brugger SP, Howes OD. Heterogeneity and homogeneity of regional brain structure in schizophrenia: a meta-analysis. JAMA Psychiatry. 2017;74:1104–11. 28973084 10.1001/jamapsychiatry.2017.2663 PMC5669456

[75] O’Neill A, Mechelli A, Bhattacharyya S. Dysconnectivity of large-scale functional networks in early psychosis: a meta-analysis. Schizophr Bull. 2019;45:579–90. 29982729 10.1093/schbul/sby094 PMC6483589

[76] Czepielewski LS, Wang L, Gama CS, Barch DM. The relationship of intellectual functioning and cognitive performance to brain structure in schizophrenia. Schizophr Bull. 2017;43:355–64. 27369471 10.1093/schbul/sbw090 PMC5605271

[77] Caldiroli A, Buoli M, van Haren NEM, de Nijs J, Altamura AC, Cahn W. The relationship of IQ and emotional processing with insula volume in schizophrenia. Schizophr Res. 2018;202:141–8. 29954697 10.1016/j.schres.2018.06.048

[78] Kang N, Chung S, Lee SH, Bang M. Cerebro-cerebellar gray matter abnormalities associated with cognitive impairment in patients with recent-onset and chronic schizophrenia. Schizophrenia (Heidelb). 2024;10:11. 38280893 10.1038/s41537-024-00434-8 PMC10851702

[79] Dempster K, Norman R, Theberge J, Densmore M, Schaefer B, Williamson P. Cognitive performance is associated with gray matter decline in first-episode psychosis. Psychiatry Res Neuroimaging. 2017;264:46–51. 28458083 10.1016/j.pscychresns.2017.04.007

[80] Bernard JA, Russell CE, Newberry RE, Goen JR, Mittal VA. Patients with schizophrenia show aberrant patterns of basal ganglia activation: Evidence from ALE meta-analysis. Neuroimage Clin. 2017;14:450–63. 28275545 10.1016/j.nicl.2017.01.034 PMC5328905

[81] Kohler S, Wagner G, Bar KJ. Activation of brainstem and midbrain nuclei during cognitive control in medicated patients with schizophrenia. Hum Brain Mapp. 2019;40:202–13. 30184301 10.1002/hbm.24365 PMC6865428

[82] Howes O, Marcinkowska J, Turkheimer FE, Carr R. Synaptic changes in psychiatric and neurological disorders: state-of-the art of in vivo imaging. Neuropsychopharmacology. 2024;50:164–83. 39134769 10.1038/s41386-024-01943-x PMC11525650

[83] Cropley VL, Klauser P, Lenroot RK, Bruggemann J, Sundram S, Bousman C, et al. Accelerated gray and white matter deterioration with age in schizophrenia. Am J Psychiatry. 2017;174:286–95. 27919183 10.1176/appi.ajp.2016.16050610

[84] Vita A, De Peri L, Deste G, Barlati S, Sacchetti E. The effect of antipsychotic treatment on cortical gray matter changes in schizophrenia: does the class matter? a meta-analysis and meta-regression of longitudinal magnetic resonance imaging studies. Biol Psychiatry. 2015;78:403–12. 25802081 10.1016/j.biopsych.2015.02.008

[85] Bennett MR. Schizophrenia: susceptibility genes, dendritic-spine pathology and gray matter loss. Prog Neurobiol. 2011;95:275–300. 21907759 10.1016/j.pneurobio.2011.08.003

[86] Howes OD, Cummings C, Chapman GE, Shatalina E. Neuroimaging in schizophrenia: an overview of findings and their implications for synaptic changes. Neuropsychopharmacology. 2023;48:151–67. 36056106 10.1038/s41386-022-01426-x PMC9700830

