# Supplementary material for: Whole brain grey matter synaptic terminal density, age and intellectual functioning in schizophrenia: an in vivo [11C]UCB-J positron emission tomography study
Source: Neuropsychopharmacology. 2026 Feb 3;51(6):1023–31. doi: 10.1038/s41386-026-02349-7 (PMC13125299; doi:10.1038/s41386-026-02349-7)
Supplement: Supplementary file 1 — Whole brain grey matter synaptic terminal density, age and intellectual functioning in schizophrenia: an in vivo [11C]UCB-J positron emission tomography study. Supplemental Material [file 41386_2026_2349_MOESM1_ESM.docx]

**Whole brain grey matter synaptic terminal density, age and intellectual functioning in schizophrenia: an in vivo [^11^C]UCB-J positron emission tomography study**

**Supplemental Material**

Ellis Chika Onwordi PhD^[[1]](#footnote-1),^^[[2]](#footnote-2),^^[[3]](#footnote-3)^^,^^[[4]](#footnote-4)^

Thomas Whitehurst PhD^1,2,4^

Ekaterina Shatalina PhD^1,2^

Ayla Mansur PhD^[[5]](#footnote-5)^

Atheeshaan Arumuham MBChB^1,2,3^

Martin Osugo PhD^1,2,3^

Tiago Reis Marques PhD^1,2^

Sameer Jauhar PhD^[[6]](#footnote-6)^

Ravi Mehrotra MRCPsych^[[7]](#footnote-7)^

Maja Ranger MD^[[8]](#footnote-8)^

Nikola Rahaman MRCPsych^[[9]](#footnote-9)^

Steve M Church MRCPsych^3^

Eugenii A. Rabiner FRCPsych SA^[[10]](#footnote-10)^^,^^[[11]](#footnote-11)^

Roger N. Gunn PhD^10,^^[[12]](#footnote-12)^

Sridhar Natesan PhD^1,2^

Abraham Reichenberg PhD^[[13]](#footnote-13)^^,^^[[14]](#footnote-14)*^

Oliver D. Howes PhD^1,2,3*^

Corresponding authors: [e.onwordi@lms.mrc.ac.uk](mailto:e.onwordi@lms.mrc.ac.uk) and [oliver.howes@kcl.ac.uk](mailto:oliver.howes@kcl.ac.uk)

*These authors contributed equally

Short title: SV2A, age and intellectual functioning in schizophrenia

**Supplementary Material and Methods**

Exclusion criteria

HVs were excluded if they had a history of a mental disorder or family history of SCZ. Volunteers were excluded from either group if they had a history of head trauma resulting in a loss of consciousness; neurological disorder; significant medical disorder; drug or alcohol dependence (except nicotine dependence); contraindications to imaging; or were taking drugs known to interact with SV2A (e.g. levetiracetam, brivaracetam, loratadine or quinine ([1](#_ENREF_1))).

Cognitive Measures

The Wechsler Adult Intelligence Scale, shortened version (WAIS-IV SF) was used to measure current intellectual functioning ([2](#_ENREF_2)). This comprised four subtests. The digit symbol substitution subtest involved first presenting the participant with nine symbol-digit pairs. Following this, the participant was asked to write the corresponding symbols underneath a list of digits rapidly, completing as much of the task as possible within the 120-second time limit. The arithmetic subtest consisted of a set of progressively difficult arithmetic questions that were read aloud to the participant who was asked to answer, testing attention, concentration, mental manipulation, and numerical reasoning ability. The test was terminated following two wrong answers. The overall number of accurate responses was recorded as the final score. The information subtest consisted of a set of general knowledge questions read aloud to the participant. The test was terminated following two consecutive incorrect responses. The overall number of accurate responses was recorded as the final score. The block design subtest tested spatial visualisation and motor skill. The participant was presented with various patterns, following which they were required to manually rearrange a set of cubes to recapitulate the given pattern. Accurate responses achieved within the time limits were recorded.

Magnetic Resonance Imaging

To facilitate the delineation of anatomical regions of interest (ROIs), we acquired T1-weighted three-dimension magnetisation prepared rapid acquisition gradient echo images from each subject, using a Siemens Magnetom Prisma 3T scanner (Siemens, Erlangen, Germany) according to the following parameters: repetition time = 2300.0 ms, echo time = 2.28 ms, flip angle = 9 °, field of view = 256 × 256 mm, 176 sagittal slices of 1-mm thickness, voxel size = 1.0 × 1.0 × 1.0 mm.

Arterial Blood Sampling

Arterial blood sampling was used throughout each PET scan to calculate the arterial input function ([3](#_ENREF_3)). Whole blood activity for the first 15 minutes was measured using a continuous automatic blood sampling system (Allogg AB, Mariefred, Sweden), and discrete samples were taken at 10, 15, 20, 25, 30, 40, 50, 60, 70, 80 and 90 minutes after tracer injection. We used a Perkin Elmer 1470 10-well gamma counter to measure plasma radioactivity and total blood concentrations, and high-performance liquid chromatography to measure the plasma radioactivity fraction constituted by unchanged parent radioligand from discrete blood samples. We measured the plasma free fraction by ultrafiltration in triplicate using an arterial blood sample taken before tracer injection.

**Supplementary Results**

Normality of data distribution

Age in the HV (KS = 0.20, *p* = 0.01) and SCZ group (KS = 0.14, *p* = 0.03), duration of illness in the SCZ group (KS = 0.24, *p* < 0.001), chlorpromazine-equivaent antipsychotic drug dose in the SCZ group (KS = 0.24, *p* = 0.005), NART-IQ in the HV (KS = 0.19, *p* = 0.045) and SCZ group (KS = 0.22, *p* < 0.001), injected radioactivity in the SCZ group (KS = 0.21, *p* < 0.001), plasma free-fraction in the SCZ group (KS = 0.19, *p* < 0.001), and CS *V*_T_ (KS = 0.17, *p* = 0.002) were not normally distributed.

*Association between whole brain grey matter [^11^C]UCB-J DVR and injected radioactivity*

Given that injected radioactivity was significantly greater in the healthy volunteer group compared to the schizophrenia group, we conducted exploratory analyses testing for associations between this and whole brain grey matter [^11^C]UCB-J DVR. There were no significant associations between injected radioactivity and whole brain grey matter [^11^C]UCB-J DVR in the combined sample (rho = 0.24, *p* = 0.12, *n* = 69), HV (rho = 0.07; *p* = 0.74, *n* = 26) or SCZ group (rho = 0.21; *p* = 0.27, *n* = 43).

We performed an ANCOVA to determine the effect of group on whole brain grey matter [^11^C]UCB-J DVR whilst covarying for injected radioactivity. After controlling for injected radioactivity, there a remained a significant effect of group on whole brain grey matter [^11^C]UCB-J DVR (*F*_1,66_ = 4.52, *p* = 0.04, η^2^ = 0.06). There was no significant effect of injected radioactivity on whole brain grey matter [^11^C]UCB-J DVR (*F*_1,66_ = 1.06, *p* = 0.31, η^2^ = 0.02).

*Association between whole brain grey matter [^11^C]UCB-J DVR and chlorpromazine-equivalent antipsychotic dose*

In the antipsychotic-treated SCZ subgroup, chlorpromazine-equivalent antipsychotic dose was not significantly correlated with whole brain grey matter [^11^C]UCB-J DVR (rho = -0.37, *p* = 0.44, *n* = 20, Supplementary Figure 1). Chlorpromazine-equivalent antipsychotic dose was not associated with WAIS-IQ (rho = -0.26, *p* = 0.96, *n* = 9), NART-IQ (rho = 0.09, *p* = 0.96, *n* = 14), or NART-WAIS IQ difference score (rho = 0.02, *p* = 0.96, *n* = 8).

Partial correlations controlling for the effect of chlorpromazine-equivalent antipsychotic dose revealed no significant correlations between whole brain grey matter [^11^C]UCB-J DVR and WAIS-IQ (*r* = 0.04, *p* = 0.93), NART-IQ (*r* = -0.13, *p* = 0.93), or NART-WAIS IQ difference score (*r* = -0.13, *p* = 0.93).


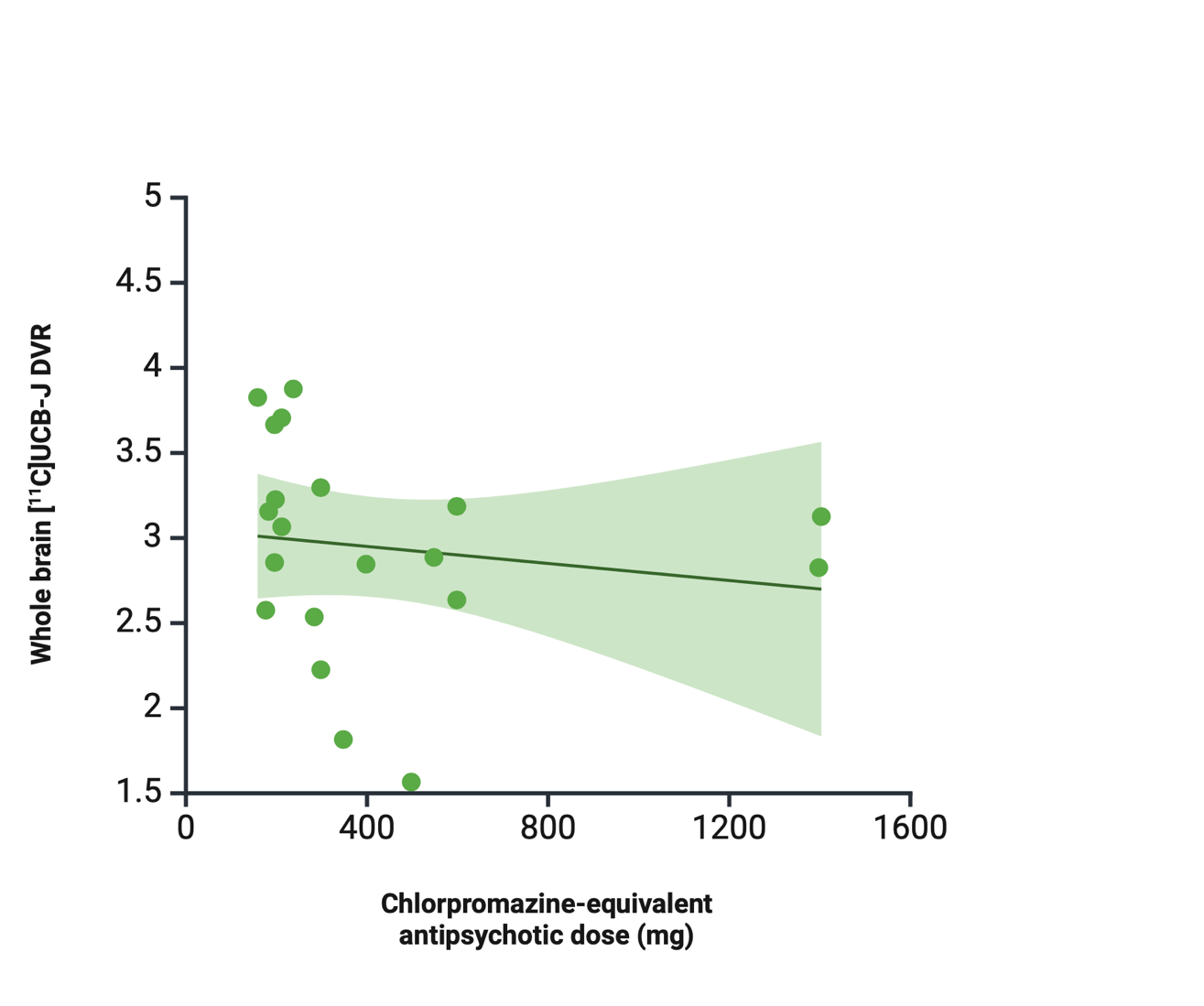


Supplementary Figure 1 Association between whole brain grey matter [^11^C]UCB-J DVR and chlorpromazine-equivalent antipsychotic dose in the medicated schizophrenia subgroup. There was no significant relationship between [^11^C]UCB-J DVR and chlorpromazine-equivalent antipsychotic dose (rho = -0.38, FDR-corrected p = 0.44). Linear regression line shown. Shaded area indicates 95% confidence interval.

*Group comparison of whole brain grey matter [^11^C]UCB-J DVR excluding cannabis users*

We conducted a sensitivity analysis excluding subjects reporting cannabis use within the past month in both HV (*n* = 1) and SCZ (*n* = 4) groups. Mean [SEM] whole brain grey matter [^11^C]UCB-J DVR remained significantly lower in SCZ group (3.04 [0.10] compared to the HV group (3.41 [0.09], *t* = 2.67, *p* = 0.01, Cohen’s *d* = 0.67).

*Associations between [^11^C]UCB-J DVR and intellectual functioning measures controlling for age*

Given the correlations between age and SV2A binding, we conducted partial correlations between [^11^C]UCB-J DVR and intellectual functioning measures, controlling for the effect of age. We found no significant association between whole brain grey matter [^11^C]UCB-J DVR and WAIS-IQ in the combined sample (*r* = 0.12, *p* = 0.44), HV group (*r* = 0.25, *p* = 0.44) or SCZ group (*r* = -0.24, *p* = 0.44). We found no significant association between [^11^C]UCB-J DVR and NART-IQ in the combined sample, (*r* = 0.13, *p* = 0.87), HV group (*r* = 0.04, *p* = 0.87), or SCZ group (*r* = -0.10, *p* = 0.87). We found no significant association between [^11^C]UCB-J DVR and NART-WAIS IQ difference score in the combined sample (*r* = -0.08, *p* = 0.92), HV group (*r* = -0.18, *p* = 0.92) or SCZ group (*r* = -0.02, *p* = 0.94).

Supplementary Table 1 Correlations between whole brain grey matter [^11^C]UCB-J DVR and WAIS subtests in the combined sample, healthy volunteer (HV) and schizophrenia (SCZ) groups.

|  | | **Combined sample** | | **HV** | | **SCZ** | |
| --- | --- | --- | --- | --- | --- | --- | --- |
|  |  | Rho | P value | Rho | P value | Rho | P value |
| **WAIS subtests** | Digit symbol coding | 0.22 | 0.22 | 0.46 | 0.16 | 0.49 | 0.88 |
|  | Arithmetic | 0.24 | 0.22 | 0.50 | 0.16 | 0.005 | 0.98 |
|  | Block design | -0.24 | 0.22 | -0.34 | 0.23 | -0.19 | 0.48 |
|  | Information | -0.09 | 0.65 | 0.36 | 0.22 | -0.41 | 0.16 |

Supplementary Table 2 Correlations between intellectual functioning measures and frontal and temporal [^11^C]UCB-J DVR in the combined sample, healthy volunteer (HV) and schizophrenia (SCZ) groups.

|  |  | **Combined sample** | | **HV** | | **SCZ** | |
| --- | --- | --- | --- | --- | --- | --- | --- |
|  |  | Rho | P value | Rho | P value | Rho | P value |
| **Frontal cortex [^11^C]UCB-J DVR** | **WAIS-IQ** | 0.08 | 0.60 | 0.27 | 0.45 | -0.22 | 0.45 |
|  | **NART-IQ** | 0.20 | 0.39 | 0.003 | 0.99 | 0.20 | 0.39 |
|  | **NART-WAIS IQ difference score** | 0.03 | 0.85 | -0.27 | 0.41 | 0.37 | 0.27 |
| **Temporal lobe [^11^C]UCB-J DVR** | **WAIS-IQ** | 0.15 | 0.34 | 0.36 | 0.34 | -0.23 | 0.34 |
|  | **NART-IQ** | 0.21 | 0.39 | 0.12 | 0.61 | 0.12 | 0.50 |
|  | **NART-WAIS IQ difference score** | -0.07 | 0.68 | -0.33 | 0.36 | 0.26 | 0.36 |

References

1. Danish A, Namasivayam V, Schiedel AC, Muller CE. Interaction of Approved Drugs with Synaptic Vesicle Protein 2A. Arch Pharm (Weinheim). 2017;350(3-4).

2. Bulzacka E, Meyers JE, Boyer L, Le Gloahec T, Fond G, Szoke A, et al. WAIS-IV Seven-Subtest Short Form: Validity and Clinical Use in Schizophrenia. Arch Clin Neuropsychol. 2016;31(8):915-25.

3. Mansur A, Rabiner EA, Comley RA, Lewis Y, Middleton LT, Huiban M, et al. Characterization of 3 PET tracers for Quantification of Mitochondrial and Synaptic function in Healthy Human Brain: (18)F-BCPP-EF, (11)C-SA-4503, (11)C-UCB-J. J Nucl Med. 2019.

1. Department of Psychosis Studies, Institute of Psychiatry, Psychology & Neuroscience, King’s College London, London SE5 8AF, UK. [↑](#footnote-ref-1)
2. MRC Laboratory of Medical Sciences, Imperial College London, London, UK [↑](#footnote-ref-2)
3. South London and Maudsley NHS Foundation Trust, Camberwell, London SE5 8AF, UK. [↑](#footnote-ref-3)
4. East London NHS Foundation Trust, 9 Alie St, London E1 8DE [↑](#footnote-ref-4)
5. IQVIA [↑](#footnote-ref-5)
6. Division of Psychiatry, Imperial College London, 2nd Floor, Commonwealth Building, Du Cane Road, London, W12 0NN [↑](#footnote-ref-6)
7. Lakeside Unit, West Middlesex University Hospital, West London NHS Trust, Twickenham Road, Isleworth, London TW7 6AF [↑](#footnote-ref-7)
8. Westminster Community Rehabilitation Team & Bluebell Lodge, Central and North West London NHS Foundation Trust, 7A Woodfield Road, London W9 2NW [↑](#footnote-ref-8)
9. Kensington Chelsea and Westminster Early Intervention Service, Central and North West London NHS Foundation Trust, Hathaway House, 7F Woodfield Road, London W9 2BA [↑](#footnote-ref-9)
10. Invicro, Burlington Danes Building, Du Cane Road, London W12 0NN, UK. [↑](#footnote-ref-10)
11. Centre for Neuroimaging Sciences, Institute of Psychiatry, Psychology and Neuroscience, King’s College London, De Crespigny Park, London SE5 8AF, UK. [↑](#footnote-ref-11)
12. Department of Brain Sciences, Imperial College London, The Commonwealth Building, Hammersmith Hospital, Du Cane Road, London W12 0NN, UK. [↑](#footnote-ref-12)
13. Department of Psychiatry, Icahn School of Medicine at Mount Sinai, New York, NY, 10029, United States [↑](#footnote-ref-13)
14. Department of Environmental Medicine and Public Health, Icahn School of Medicine at Mount Sinai, New York, NY, 10029, United States  [↑](#footnote-ref-14)
